# Diagnostic Performance of PCR-Based Detection of Human Herpesvirus 8 DNA in Archived Formalin Fixed Paraffin Embedded Biopsy Specimens of Kaposi Sarcoma

**DOI:** 10.3390/v18060623

**Published:** 2026-05-29

**Authors:** Cemal Çiçek, Efe Yetişgin, Ayfer Bakır, Elif Tuğçe Güner, Murat Alper, Murat Aral

**Affiliations:** 1 Department of Medical Microbiology, University of Health Science Ankara Etlik City Hospital, Ankara 06170, Türkiye; dr.ayfer.bakir@gmail.com (A.B.); eliftugce06md@gmail.com (E.T.G.); aralmurat@hotmail.com (M.A.); 2 Department of Medical Pathology, University of Health Science Ankara Etlik City Hospital, Ankara 06170, Türkiye; 89efe89@gmail.com (E.Y.); muratalper70@gmail.com (M.A.)

**Keywords:** Kaposi sarcoma, human herpesvirus 8, polymerase chain reaction, immuno-histochemistry, sensitivity, specificity

## Abstract

Background: Kaposi sarcoma (KS) is a vascular malignancy closely associated with human herpesvirus 8 (HHV-8) infection, and its diagnosis relies on combined clinical, histopathological and molecular assessment. This study aimed to detect HHV-8 DNA in archived formalin fixed paraffin embedded (FFPE) biopsy samples of KS by real time PCR and to evaluate the diagnostic performance of PCR compared with histopathology. Meth ods: In this retrospective cross-sectional study, 98 FFPE biopsy specimens with histopathologically confirmed KS and 30 FFPE biopsy specimens with non-KS vascular lesions were included as the patient and control groups, respectively. HHV-8 DNA was analyzed in all samples using real-time PCR. Diagnostic performance parameters, including sensitivity, specificity, predictive values, and accuracy, were calculated, and agreement with histopathological diagnosis was assessed using Cohen’s kappa coefficient. Results: HHV-8 PCR was positive in 89.8% (88/98) of KS cases and negative in all controls (30/30; 100%). Sensitivity, specificity, positive predictive value, negative predictive value and overall accuracy were 89.8%, 100%, 100%, 75.0% and 92.2%, respectively. Overall agreement between PCR and histopathology was 92.2% with a Cohen’s kappa of 0.34 (*p* < 0.001), indicating fair concordance. PCR positivity rates and cycle threshold values did not differ significantly between histopathological stages, or according to histopathological and immunohistochemical parameters. Conclusions: HHV-8 PCR in archived FFPE KS biopsies shows high specificity and good sensitivity and acts as a complementary diagnostic tool with fair agreement with histopathology. It is particularly valuable in diagnostic gray areas but should always be interpreted together with clinical, histopathological and immunohistochemical findings.

## 1. Introduction

Kaposi sarcoma (KS) is an HHV-8-associated angioproliferative neoplasm originating from endothelial lineage cells [1,2]. HHV-8, also known as Kaposi’s sarcoma-associated herpesvirus (KSHV), is a double-stranded DNA virus belonging to the *Gammaherpesvirinae* subfamily of the *Herpesviridae* family and the genus *Rhadinovirus* [3]. HHV-8 is a ‘Group 1 carcinogen’ and is the main causative agent of KS and some lymphoproliferative diseases [4]. The seroprevalence of HHV-8 shows significant geographical variation; it is over 50% in Sub-Saharan Africa, between 10 and 30% in the Mediterranean basin, Eastern Europe, and the Caribbean, and below 10% in Northern Europe, Asia, and the USA [5]. While HHV-8 is mainly transmitted through saliva, sexual contact, blood transfusion, and organ transplantation are other important transmission routes [4]. HHV-8 persists in endothelial cells through latent and lytic phases. In the latent phase, the LANA-1 protein ensures the persistence of the virus in the host, while proteins such as vGPCR and viral IL-6 contribute to neoplastic transformation by increasing cell proliferation, angiogenesis, and inflammation [2].

The most important risk factor for the development of KS is HHV-8 infection, and the onset of the disease is mostly associated with immune system suppression [6]. KS is most commonly seen in the skin and mucous membranes, especially in the lower extremities and oral mucosa. It can also involve internal organs such as lymph nodes, the gastrointestinal system, and the lungs. More rarely, the musculoskeletal system, bones, eyes, endocrine organs, and heart may be affected. As involvement becomes widespread, the disease progresses more aggressively [7,8]. KS appears in four clinical forms: classic, endemic, iatrogenic, and epidemic. The classic form occurs in elderly Mediterranean men, the endemic form in Africa, the iatrogenic form with immunosuppression, and the epidemic form in association with HIV [9,10,11,12]. KS shows a progressive pattern consisting of patch, plaque, and nodular stages. The main findings are vascular proliferation, spindle cell increase, and slit-like vascular structures. Different histological variants can also be observed [12,13,14].

KS can be confused clinically and histopathologically with benign and malignant vascular lesions. Particularly with angiosarcoma and bacillary angiomatosis, and in the early stage with benign vascular lesions, differential diagnosis can be difficult. Therefore, an accurate diagnosis is of critical clinical importance [10,14,15]. Accurate diagnosis of KS requires integration of clinical presentation with histopathological, immunohistochemical (IHC), and molecular data [10,15]. In diagnosis, LANA-1 immunohistochemical staining is the gold standard, and detection of HHV-8 DNA by PCR, especially in suspicious cases, supports the diagnosis [10,12,15].

Although HHV-8 PCR has previously been investigated in FFPE tissues, many earlier studies were limited by small sample sizes, heterogeneous PCR methodologies, lack of standardized commercial real-time PCR assays, and insufficient evaluation of analytical and diagnostic performance parameters such as internal quality controls, sensitivity, and specificity. In addition, comparative data obtained from clinically characterized FFPE Kaposi sarcoma samples and histopathological control groups remain limited. Therefore, further validation studies using standardized real-time PCR assays in routine clinical FFPE samples are needed.

Accordingly, the present study aimed to evaluate the diagnostic performance of a commercially available real-time PCR assay targeting HHV-8 DNA in a relatively large cohort of archived FFPE KS biopsy specimens and to compare the molecular findings with histopathological diagnosis.

## 2. Materials and Methods

### 2.1. Study Design

The study was designed as a retrospective, cross-sectional methodological study. The study population consisted of patients who underwent biopsy for suspected KS between 1 January 2023, and 1 January 2026, at various clinics of the hospital. HHV-8 DNA was analyzed by real-time PCR using archived formalin-fixed, paraffin-embedded (FFPE) tissue biopsy specimens. The sample size was determined based on all available data due to the retrospective archive-based design of the study, and a prospective power calculation could not be performed. This should be considered a limitation of the study.

### 2.2. Study Population and Sample Selection

This study included tissue samples that were biopsied with suspicion of KS, sent to the pathology laboratory, and preserved as FFPE specimens. The patient group consisted of 98 cases in which KS was confirmed by histopathological examination, while the control group was formed from 30 FFPE samples that were biopsied with suspicion of KS but were histopathologically excluded for KS and diagnosed with vascular lesions such as angioma, pyogenic granuloma, hemangioma, and bacillary angiomatosis. These vascular lesions were included in the control group because they may clinically and histopathologically mimic KS and are relevant in the differential diagnosis of KS.

### 2.3. Inclusion and Exclusion Criteria

Patients aged 18 years and older who were clinically suspected of KS, had undergone biopsy, and had sufficient archived FFPE tissue material available for molecular analysis were included in the study. If multiple samples from the same patient were available, only the first sample was included. Non-vascular malignancies, metastatic lesions, post-treatment biopsy specimens, and samples with insufficient tissue for molecular analysis were excluded from the study.

### 2.4. Histopathological Evaluation

Biopsy samples of KS-suspected cases included in the study were fixed in 10% buffered formalin and embedded in paraffin blocks according to standard procedures. Sections 4–5 µm thick obtained from these blocks were evaluated with routine Hematoxylin–Eosin (H&E) staining. All tissue sections were retrospectively re-examined under a light microscope (Olympus BX51, Tokyo, Japan) by an experienced pathologist (E.Y.), independently of clinical and molecular data.

In the histopathological evaluation, established morphological criteria described in the literature were used as the basis [16]. The existing preparations were re-evaluated in terms of characteristic findings, primarily spindle cell proliferation, slit-like vascular structures, and erythrocyte extravasation; the conformity of the current diagnoses with these morphological criteria was reviewed. HHV-8 LANA-1, CD31, CD34, and Ki-67 IHC staining results were obtained from archived pathology reports and evaluated together with the histopathological findings. Following histopathological evaluation, representative FFPE tissue areas containing adequate lesional tissue and suitable for molecular analysis were selected for DNA extraction and PCR analysis.

### 2.5. HHV-8 DNA Extraction and Real-Time PCR Analysis

All necessary precautions were taken during the tissue preparation stage to prevent contamination. From FFPE tissue blocks, two 5 µm-thick sections were obtained from each sample using sterile, single-use scalpels and transferred into 1.5 mL screw-cap tubes. The obtained sections were stored at room temperature until further processing. For deparaffinization, 1000 µL of xylene was added to each tube, and the samples were incubated at 45 °C for 15 min before centrifugation at 10,000 rpm. After removing the supernatant, the same process was repeated with fresh xylene. Then, to remove xylene residues, the pellet was washed twice with 98% ethanol. After the ethanol was removed, the samples were left to dry completely and then the DNA isolation stage was initiated.

Before DNA isolation, the samples were pre-processed using the Zybio EXM3000 (Zybio Inc., Chongqing, China) automatic extraction system. Subsequently, DNA isolation was carried out using the Biospeedy^®^ Rapid Nucleic Acid Extraction Kit developed by Bioeksen (Bioeksen R&D Technologies Inc., Istanbul, Türkiye) according to the manufacturer’s instructions. DNA was eluted in a final volume of 50 µL using the elution buffer provided in the kit.

The concentration and purity of the extracted DNA were measured using a NanoDrop 2000 spectrophotometer (Thermo Fisher Scientific, Wilmington, DE, USA), and DNA suitability for PCR analysis was assessed. Approximately 100 ng of DNA was used for each PCR reaction. Extracted DNA samples were stored at −80 °C until real-time PCR analysis. DNA quality and extraction adequacy were evaluated by amplification of the human RNase P internal control gene during real-time PCR analysis. Successful RNase P amplification indicated adequate nucleic acid recovery and the presence of amplifiable human DNA in the samples.

Extracted genomic DNA samples were analyzed using a real-time PCR assay targeting HHV-8-specific genomic regions. The amplification processes were carried out using a commercial HHV-8 real time PCR kit (Bioeksen R&D Technologies, Istanbul, Türkiye; catalog no: BS-HHV8-25) according to the manufacturer’s instructions.

PCR reactions were performed using the Magnetic Induction Cycler (MIC)-PCR system (Bio Molecular Systems, Upper Coomera, Australia). The test system used was designed to target the latent nuclear antigen (LANA, ORF73) gene region in the HHV-8 genome, thereby aiming to detect the virus even in its latent phase.

Each PCR reaction consisted of 10 µL of reaction mix and 10 µL of extracted DNA, resulting in a final reaction volume of 20 µL. Thermal cycling conditions consisted of enzyme activation at 52 °C for 3 min, followed by initial denaturation at 95 °C for 10 s, 12 touchdown cycles of 95 °C for 1 s and 67–56 °C for 15 s, and 30 amplification cycles of 95 °C for 1 s and 55 °C for 15 s with fluorescence detection in the FAM and HEX channels.

Fluorescent signals generated during the amplification process were monitored using Sigmoida Software (V8.6 REV.56), and cycle threshold (Ct) values were automatically calculated. For result interpretation, sigmoidal amplification curves and Ct values below the threshold were accepted as positivity criteria.

Each PCR run included a manufacturer-provided positive control, a no-template negative control, and negative extraction controls to monitor contamination during both amplification and extraction procedures. Samples were considered valid only when amplification of the human RNase P gene used as an internal control was within the expected range.

According to the manufacturer’s specifications, the PCR kit has a sensitivity of 100% and a specificity of 99.68%. In addition, the limit of detection (LoD) ranges between 140.87 and 175.99 copies/µL depending on the specimen type.

### 2.6. Statistical Analysis

Statistical analysis of the obtained data was performed using SPSS software (version 27.0; IBM Corp., Armonk, NY, USA). Continuous variables were expressed as median and interquartile range (IQR) according to their distribution characteristics, and categorical variables were expressed as number (*n*) and percentage (%). For comparisons between groups, the Pearson chi-square test or Fisher’s exact test, where appropriate, was used for categorical variables. In the comparison of continuous variables, the Mann–Whitney U test was preferred when the assumption of normal distribution was not met, and the Kruskal–Wallis test was used for comparisons of more than two groups. The relationships between HHV-8 PCR positivity and demographic and clinical variables were evaluated using appropriate non-parametric tests. The relationship between Ct values and histopathological parameters and IHC findings was examined using Spearman correlation analysis. The diagnostic performance of the PCR method was evaluated by calculating sensitivity, specificity, positive predictive value (PPV), negative predictive value (NPV), and accuracy. The agreement between PCR results and histopathological diagnosis was analyzed using Cohen’s kappa (κ) coefficient. In all statistical analyses, a *p*-value of <0.05 was considered statistically significant.

## 3. Results

### 3.1. Clinical Features of Kaposi Sarcoma Cases

The hospital information management system records of 98 patients diagnosed with KS were retrospectively examined. Of the cases, 65.3% were male (*n* = 64) and 34.7% were female (*n* = 34), and the vast majority of patients were aged 50 years and older (89.8%, *n* = 88). The most common location of lesions was the skin (91.8%; *n* = 90), with 82.7% of lesions (*n* = 81) occurring in a single anatomical region and 17.3% (*n* = 17) in multiple regions. Single lesions were most frequently observed in the lower extremities (64.3%; *n* = 63), while multiple lesions were mostly seen as combinations involving extremities together (Table 1).

The majority of patients had no comorbidity (63.3%), while metabolic diseases were the most common comorbidity (23.5%), predominantly diabetes mellitus. Other comorbidities, including malignancies (6.1%), autoimmune diseases (4.1%), and immune deficiencies, were less frequent. The distribution of comorbidities in KS patients is presented in Table 2.

### 3.2. Clinical and Lesion Characteristics of the Control Group

The median age of the 30 cases in the control group was determined to be 42 years (IQR: 32–48). Of the total 30 control cases who underwent biopsy due to suspected KS and were histopathologically negative, 53.3% were male (*n* = 16). It was determined that 23.3% of the control cases were under 30 years of age (*n* = 7), 53.3% were between 30 and 50 years old (*n* = 16), and 23.3% were 50 years and older (*n* = 7). The localization distribution of lesions suspected of KS was as follows: head 30.0% (*n* = 9), upper extremity 26.7% (*n* = 8), trunk 20.0% (*n* = 6), lower extremity 6.7% (*n* = 2), and neck 3.3% (*n* = 1); oral mucosa 6.7% (*n* = 2) and nasal mucosa 6.7% (*n* = 2). Only one case (3%) in the control group was found to have diabetes mellitus as a comorbidity.

### 3.3. Histopathological and Immunohistochemical Characteristics of Kaposi Sarcoma Cases

In histopathological evaluation, the most common HHV-8 immunohistochemical expression level was 70%, followed by 60%. The median HHV-8 immunohistochemical expression level was 70% in both PCR-positive and PCR-negative cases, with no statistically significant difference between the groups (*p* = 1.00). All histopathologically confirmed KS cases showed LANA-1 positivity, whereas all control cases were LANA-1 negative. Among the 98 LANA-1-positive KS cases, HHV-8 PCR was positive in 88 cases and negative in 10 cases.

**Figure 1:**
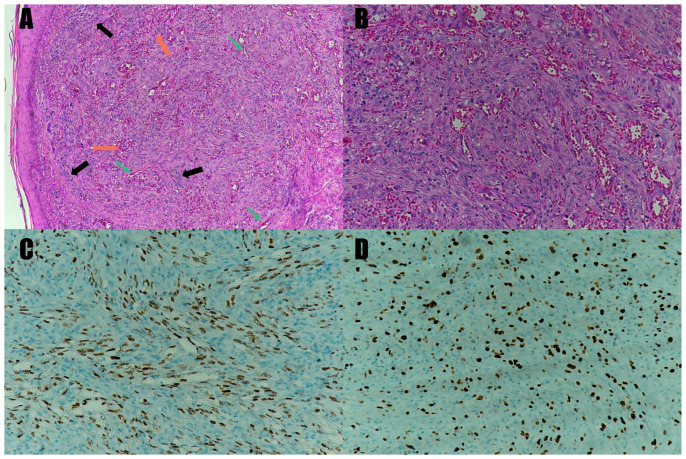
Histopathological and immunohistochemical features of nodular-stage KS. (**A**) The nodular-stage lesion shows characteristic slit-shaped vascular spaces (green arrows), a dense inflammatory infiltrate predominantly composed of lymphocytes (black arrows), and areas of erythrocyte extravasation (orange arrows) (H&E, ×100). (**B**) At higher magnification, spindle cell proliferation forming fascicles and irregular vascular spaces are observed (H&E, ×200). (**C**) Immunohistochemical staining for HHV-8 shows diffuse nuclear positivity in neoplastic spindle cells (×200). (**D**) Ki-67 immunostaining demonstrates a high proliferative index in the tumor cells (×200).

The nodular stage was observed in 49.0% of cases, vascular slit formation in 81.6%, and erythrocyte extravasation in 84.7%. Prominent spindle cell proliferation was detected in 37.8% of the samples. Cellular atypia was most commonly of moderate intensity. The histopathological and IHC characteristics of KS patients are presented in Table 3. Representative histopathological images of nodular-stage KS are shown in Figure 1.

The storage durations of FFPE samples were compared between HHV-8-positive and HHV-8-negative groups. The median storage duration was 27 months in HHV-8-negative samples and 21 months in HHV-8-positive samples (*p* = 0.257).

When HHV-8 expression percentages were evaluated according to histopathological stages, the median was 70% (IQR: 60–80) in the patch stage, 60% (IQR: 60–70) in the plaque stage, and 70% (IQR: 60–80) in the nodular stage. There was no statistically significant difference in HHV-8 percentages between the groups (*p* = 0.543).

### 3.4. HHV-8 PCR Positivity Rates and Internal Control Values

HHV-8 PCR was positive in 89.8% of KS patients (*n* = 88; 95% CI: 81.9–94.8) and negative in 10.2% (*n* = 10; 95% CI: 5.2–18.1). In the control group, PCR results were negative in all cases (100%; *n* = 30; 95% CI: 88.6–100). Detailed HHV-8 PCR results and internal control characteristics of KS and control groups are summarized in Table 4.

**Figure 2:**
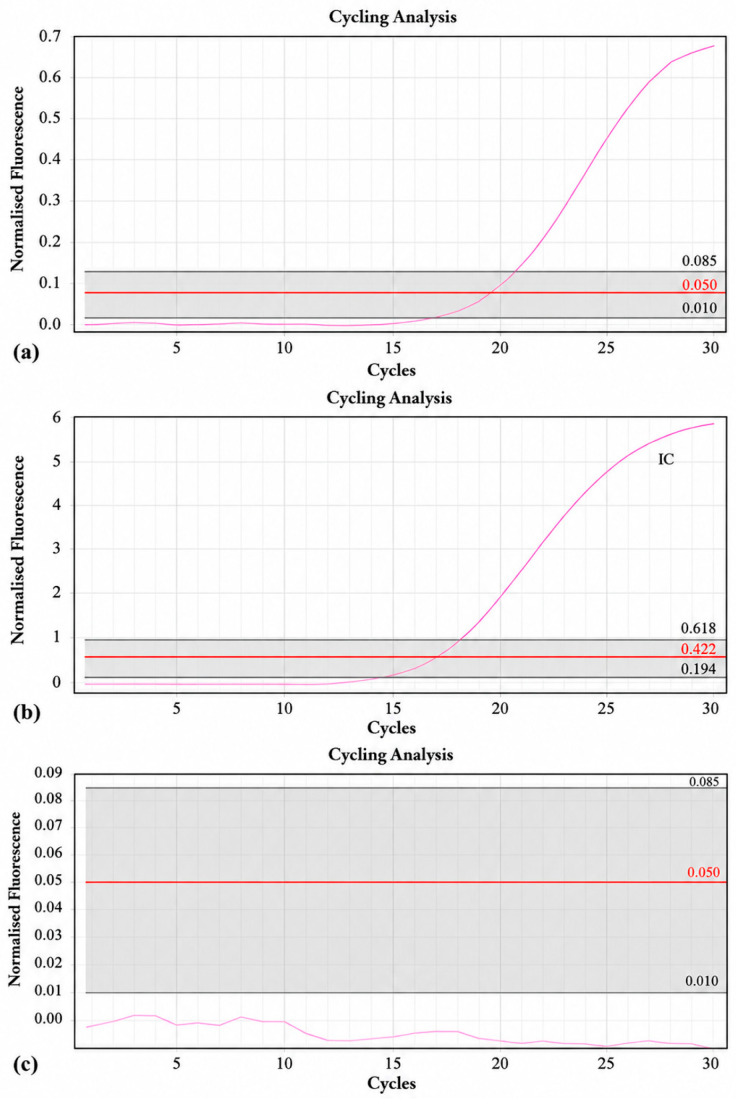
Representative real-time PCR amplification curves. (**a**) Representative HHV-8-positive patient sample showing successful amplification of the HHV-8 target sequence. (**b**) Representative internal control (RNase P) amplification curve confirming adequate nucleic acid extraction and amplification efficiency. (**c**) Representative negative control showing absence of HHV-8 amplification. Colored sigmoidal curves represent amplification curves, while grey shaded areas indicate the threshold/baseline regions used for Ct determination.

Representative real-time PCR amplification curves of an HHV-8-positive patient sample, internal control amplification, and a negative control are presented in Figure 2.

In cases with confirmed KS diagnosis, the median cycle threshold (Ct) value in HHV-8 PCR analysis was determined as 20.92 (IQR: 18.26–22.85). The median value of internal controls was calculated as 16.54 (IQR: 14.69–17.91). In samples with a negative Ct value, internal control values ranged from 13.62 to 20.82.

In samples from the control group, the median value of internal controls was calculated as 16.52 (IQR: 15.12–18.57).

### 3.5. Relationship of HHV-8 PCR Positivity with Clinical Variables

No statistically significant relationship was found between HHV-8 PCR positivity and gender (*p* = 0.303). PCR positivity was found to be 87.5% (56/64) in men and 94.1% (32/34) in women.

PCR positivity by age group was 66.7% (2/3) in the <30 age group, 100% (7/7) in the 30–50 age group, and 89.8% (79/88) in the ≥50 age group (*p* = 0.280).

PCR positivity by lesion localization was 91.1% (82/90) for skin, 100% (2/2) for lymph nodes, 50.0% (1/2) for stomach, and 75.0% (3/4) for mucosa (*p* = 0.186).

### 3.6. Diagnostic Performance of HHV-8 PCR and Correlation with Histopathology

When the diagnostic performance of HHV-8 PCR was evaluated, sensitivity was calculated as 89.8%, specificity as 100%, PPV as 100%, and NPV as 75.0%. The overall accuracy rate was determined as 92.2% (Table 5).

### 3.7. Real-Time PCR Cycle Threshold Values and Their Relationship with Histopathological Stage and Immunohistochemical Parameters

**Figure 3:**
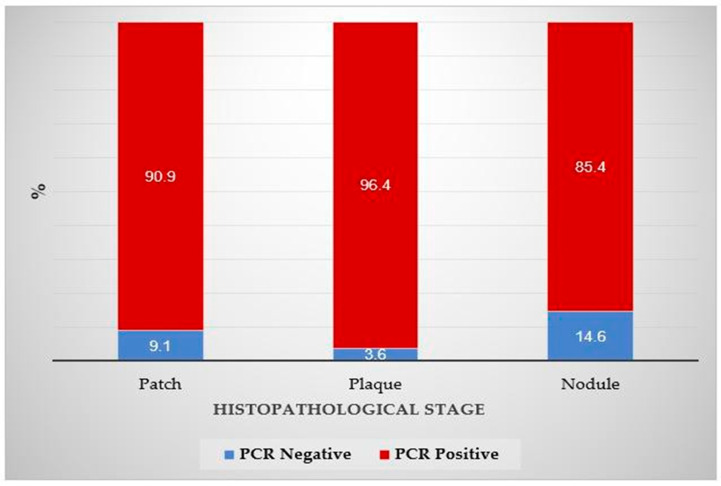
Distribution of PCR positivity according to histopathological stages.

When PCR positivity rates were evaluated according to histopathological stages, positivity was detected at a rate of 90.9% (20/22) in the patch stage, 96.4% (27/28) in the plaque stage, and 85.4% (41/48) in the nodular stage. There was no statistically significant difference in PCR positivity rates between the groups (*p* = 0.304) (Figure 3).

When compared with histopathological diagnosis, HHV-8 PCR was positive in 88 of 98 KS cases and negative in 10 cases, while no PCR positivity was observed among IHC-negative cases. The overall agreement between the two methods was 92.2%, with a Cohen’s kappa value of 0.34 (95% CI: 0.29–0.39, *p* < 0.001), indicating fair agreement (Table 6).

Ct values showed a similar distribution according to histopathological stages, and no statistically significant difference was found between the groups (*p* = 0.154). A weak negative correlation was observed between Ct values and the Ki-67 proliferation index, which was not statistically significant (Spearman rho = −0.182; *p* = 0.090). Similarly, no significant correlation was found between Ct values and the percentage of HHV-8 (Spearman rho = 0.051; *p* = 0.638). Data are presented as median (IQR).

When Ct values were evaluated according to histopathological stages, the median Ct value was 21.52 (IQR: 20.04–22.85) in the patch stage, 20.54 (IQR: 18.52–24.26) in the plaque stage, and 20.02 (IQR: 16.92–22.19) in the nodular stage. There was no statistically significant difference in Ct values between the groups (*p* = 0.207). A weak negative correlation was detected between Ct value and histopathological stage, but this relationship was not found to be statistically significant (Spearman rho = −0.19, *p* = 0.083).

## 4. Discussion

In this study, the diagnostic performance of the HHV-8 PCR method in the diagnosis of KS was evaluated, and the findings obtained were interpreted in light of the literature. The finding that HHV-8 PCR demonstrated 89.8% sensitivity, 100% specificity, and 92.2% accuracy in our study indicates that the method is a remarkable diagnostic tool, particularly due to its high specificity. The negative PCR results in all cases in the control group, the absence of false positivity, and the positive predictive value of 100% support that PCR is a strong method for distinguishing KS from vascular lesions that can mimic it [17]. The overall agreement between histopathological diagnosis and PCR was 92.2%, with a Cohen’s kappa value of 0.34, indicating fair agreement between the two methods. The relatively low Cohen’s kappa coefficient despite the high overall agreement may be explained by the imbalance between positive and negative cases in the study population, a phenomenon commonly referred to as the “kappa paradox” or “prevalence paradox.” Cohen’s kappa is influenced not only by observed agreement but also by the agreement expected by chance, which tends to increase in datasets with markedly skewed prevalence distributions. In our study, the predominance of histopathologically confirmed KS cases together with the absence of false-positive PCR results in the control group likely increased the expected chance agreement and consequently reduced the kappa value despite the high concordance between the two methods. Therefore, in studies with imbalanced group distributions, kappa values should be interpreted together with overall agreement, sensitivity, specificity, and predictive values. These findings highlight the importance of integrating HHV-8 DNA PCR into clinical decision-making, particularly in diagnostic gray areas. In cases where histomorphological features are not typical, and LANA-1 staining is weak or equivocal, the detection of HHV-8 DNA in FFPE tissues can help strengthen the diagnosis in favor of KS. However, the fact that PCR sensitivity is not optimal due to technical limitations inherent to FFPE material indicates that its use to exclude KS solely on the basis of a negative molecular test result is insufficient. Therefore, HHV-8 DNA PCR results should always be interpreted within a holistic framework that incorporates clinical, histopathological, and immunohistochemical data. Multidisciplinary tumor boards involving dermatology, infectious diseases, oncology, and pathology can facilitate this integrated approach, helping to prevent misinterpretation of false-positive or false-negative results and enabling more accurate classification of HHV-8-positive vascular lesions.

In the literature, HHV-8 DNA has been detected in the majority of KS lesions, including classic, endemic, AIDS-related, and post-transplant variants, supporting the etiological role of HHV-8 in the pathogenesis of the disease [16,18]. In our study, the demonstration of HHV-8 DNA by PCR in most histopathologically confirmed cases is consistent with these findings. However, data showing that HHV-8 DNA can also be detected in some non-KS lesions or in tumor tissues of patients with KS indicate that PCR results should not be interpreted alone and must be evaluated together with clinical and histopathological context [19,20]. Current literature also emphasizes that a multidisciplinary approach (clinical, histopathology, immunohistochemistry, and molecular analysis) is the most reliable method for the diagnosis of KS [15,21].

In the diagnosis of KS, LANA-1 immunohistochemistry is considered the gold standard method, particularly because the direct demonstration of viral protein in the nuclei of neoplastic spindle cells provides high sensitivity. However, it is also known that IHC staining can be affected by technical factors and may yield false-negative results in some cases [2,12]. In this context, PCR, especially in cases showing atypical morphology or where IHC staining is weak/negative, provides a complementary molecular evidence, thereby increasing diagnostic accuracy [7,12,22].

In our study, HHV-8 PCR was found to be negative in 10 out of 98 histopathologically confirmed KS cases (10.2%). In these cases, the HHV-8 expression detected immunohistochemically was at a level similar to that of the PCR-positive group (median 70%), suggesting that technical limitations related to FFPE tissues may have contributed to PCR negativity. However, the possibility of truly HHV-8-negative cases cannot be completely excluded. In the literature, DNA fragmentation due to formalin fixation, the formation of cross-links, and the presence of PCR inhibitors in FFPE tissues are cited as the main causes of false negatives [7,15,23]. Additionally, in early (patch) stage lesions, the low density of neoplastic spindle cells may cause the viral load to remain below detection threshold [24]. Further evaluation of PCR-negative samples using alternative molecular approaches, such as nested PCR, in situ hybridization, or sequencing-based methods, may help clarify these findings in future studies. These findings indicate that despite the limited sensitivity of PCR, immunohistochemistry plays a complementary role in diagnosis by directly demonstrating viral proteins within the tissue.

Technical limitations specific to FFPE tissues are important factors that directly affect the performance of PCR analyses. Formalin fixation causes cross-linking in DNA, leading to fragmentation and reducing the amount of amplifiable DNA. In addition, residues of solvents used during deparaffinization and rehydration processes can inhibit polymerase activity [23]. In the literature, it is recommended to prefer targets with short amplicons (<300 bp) and to run internal control genes simultaneously in order to reduce these effects [25]. In our study, the amplification of internal controls in all samples indicates that complete PCR inhibition is excluded and supports that negative results largely arise from partial DNA damage or low target copy numbers.

The difference between the sensitivity of the commercial PCR kit used as reported by the manufacturer (100%) and the sensitivity obtained in our study (89.8%) is an expected finding, considering that kit performance values are determined using fresh or optimized nucleic acid samples. DNA fragmentation specific to FFPE tissues and PCR inhibitors inevitably reduce clinical sensitivity compared to manufacturer conditions.

The findings obtained in our study indicate that clinical, histopathological, IHC, and molecular data should be evaluated together in the diagnosis of KS. In cases with typical morphological features and strong LANA-1 positivity, the diagnosis is largely certain, whereas in atypical or suspicious situations, PCR analysis plays an important complementary role. In particular, the high specificity obtained in our study suggests that PCR can be used as a strong confirmatory test in diagnostic gray areas. However, a negative PCR result is not sufficient to rule out a KS diagnosis, especially due to technical limitations specific to FFPE tissues, and in such cases, the diagnosis should be made based on histopathological and IHC findings.

When evaluated in terms of Ct values, it was observed in our study that the median Ct value was 20.92, and no significant difference was found between histopathological stages. This suggests that the viral load in tumor tissue may be relatively stable regardless of the stage of the lesion. It has been reported in the literature that tissue PCR is mostly evaluated qualitatively and that the prognostic value of viral load is more meaningful in blood samples [10,26]. Therefore, it is understood that Ct values alone are not a reliable marker for reflecting the stage of the disease or prognosis [27].

Our histopathological findings are consistent with the literature, and the predominance of the nodular phase and the high detection rates of characteristic morphological features such as vascular slit structures and erythrocyte extravasation support the typical histological spectrum of KS [2,21,24,28]. In immunohistochemical analyses, high LANA-1 expression confirms the fundamental role of HHV-8 in tumor pathogenesis, while CD34 and CD31 positivity support the endothelial origin of the tumor [12].

There are some significant limitations to the study. Its retrospective and single-center nature limits the generalizability of the findings. Most of the study group consists of classic KS cases. Therefore, the diagnostic performance of HHV-8 PCR could not be evaluated in other subtypes such as HIV-associated and post-transplant KS. The use of only FFPE tissue is an important limitation. Formalin fixation can lead to DNA fragmentation and cross-linking, reducing PCR sensitivity [7,15,23]. This may explain the 10.2% PCR negativity in our study. Furthermore, the analytical characteristics of the assay, including the manufacturer-reported specimen-dependent LoD, should be considered when interpreting PCR results obtained from archived FFPE tissues.

In addition, neither macrodissection nor laser microdissection was performed in this study. The retrospective archive-based design of the study and the use of a standard FFPE sectioning protocol were the main reasons for not applying these methods. The use of whole tissue sections may have resulted in dilution of HHV-8-positive spindle cells with surrounding non-neoplastic tissue, potentially affecting PCR sensitivity and contributing to false-negative results. Macrodissection- and laser microdissection-based approaches may improve tumor cell enrichment and increase molecular detection sensitivity in future studies. A further limitation is that comparative evaluation of different commercial HHV-8 real-time PCR assays on the same FFPE sample set could not be performed within the scope of the present study.

Another potential limitation is the possibility of cross-contamination during FFPE tissue processing and fixation procedures. Since archived tissue samples may have been processed using shared formalin, solvent, and paraffin systems, low-level contamination between specimens cannot be completely excluded. However, strict contamination control measures, including the use of sterile single-use instruments, negative extraction controls, and no-template PCR controls, were applied throughout the study, and no PCR positivity was detected in the control group. Third, the control group consists only of cases suspected of KS but histopathologically excluded. This situation does not represent all vascular lesions and may lead to selection bias. Since the majority of the study population consisted of classic KS cases, the diagnostic performance of HHV-8 PCR in endemic, HIV-associated, and post-transplant variants could not be evaluated within the scope of this study.

Furthermore, due to the retrospective, archive-based design of the study, systematic classification of cases according to the KS clinical subtype (classic, endemic, iatrogenic, or HIV-associated) was not possible. This limits the comparison of PCR performance by subtype and restricts the generalizability of the results.

Finally, clinical follow-up and long-term prognostic data are limited. Therefore, the relationship between HHV-8 and disease course could not be evaluated. The inability to correlate molecular findings with clinical outcomes has limited interpretations regarding the prognostic value of PCR.

## 5. Conclusions

The detection of HHV-8 DNA by PCR in archived FFPE biopsies provides strong molecular evidence supporting the diagnosis, especially in cases where histomorphological findings are suspicious. However, considering technical limitations and the possibility of false negatives, PCR results should not be interpreted in isolation; they must be evaluated together with clinical, histopathological, and IHC findings. In conclusion, the most reliable approach in KS diagnosis is based on multidisciplinary integration, with HHV-8 PCR positioned within this approach as a confirmatory tool with high specificity, particularly in diagnostic gray areas.

In the future, conducting multicenter, prospective studies that include not only the classical form but also endemic, HIV-associated, and post-transplant KS variants will contribute to clarifying the diagnostic value of HHV-8 PCR in different clinical subtypes, as well as the standardization and prognostic significance of PCR-based methods.

## Figures and Tables

**Table 1:** Clinical and demographic characteristics of the patient group.

Characteristics	*n*	%
Gender		
–Male	64	65.3
–Female	34	34.7
Age groups (years)		
<30	3	3.1
30–50	7	7.1
>50	88	89.8
Localization		
–Skin	90	91.8
–Lymph node	2	2.0
–Stomach	2	2.0
–Mucosa	4	4.1
Lesion involvement		
Single anatomical region	81	82.7
–Lower extremity	63	64.3
–Head (oral cavity)	4	4.1
–Upper extremity	9	9.2
–Stomach	2	2.0
–Inguinal lymph node	2	2.0
–Scrotum	1	1.0
Multiple anatomical regions	17	17.3
–Lower extremity + hip skin	7	7.1
–Lower and upper extremity	5	5.1
–Lower extremity + neck	1	1.0
–Upper extremity + head	3	3.1
–Upper extremity + shoulder skin	1	1.0

**Table 2:** Distribution of comorbidities in patients with Kaposi sarcoma (*n* = 98).

Comorbidity Status	*n*	%
Metabolic diseases	23	23.5
-Diabetes mellitus	23	
Acquired immune deficiency	2	2.0
-HIV	2	
Inherited immune deficiency	1	1.0
-Hypogammaglobülinemi	1	
Malignancies	6	6.1
-Non-Hodgkin lymphoma	3	
-Pancreatic cancer	2	
-Bladder cancer	1	
Autoimmune diseases	4	4.1
-Systemic lupus erythematosus	3	
-Autoimmune hemolytic anemia	1	
No comorbidity	62	63.3

**Table 3:** Histopathological and immunohistochemical features of Kaposi sarcoma patients (*n* = 98).

Variable	Total	PCR Positive	PCR Negative	*p*-Value
FFPE storage, months (median, IQR)	23 (13–31)	21 (13–30)	27 (17–35)	0.257
Ki-67 index, % (median, IQR)	15 (5–30)	15 (5–30)	25 (5–35)	0.410
CD34 (+), *n* (%)	28 (28.6)	28 (100)	0 (0)	0.058
CD31 (+), *n* (%)	20 (20.4)	17 (85)	3 (15)	0.422
D2-40 (+), *n* (%)	2 (2.0)	2 (100)	0 (0)	1.00
Patch stage, *n* (%)	22 (22.4)	20 (90.9)	2 (9.1)	
Plaque stage, *n* (%)	28 (28.6)	27 (96.4)	1 (3.6)	0.347
Nodular stage, *n* (%)	48 (49.0)	41 (85.4)	7 (14.6)	
Vascular slit, *n* (%)	80 (81.6)	73 (91.3)	7 (8.8)	0.385
Spindle cells—Minimal, *n* (%)	22 (22.4)	19 (86.4)	3 (13.6)	
Spindle cells—Moderate, *n* (%)	39 (39.8)	35 (89.7)	4 (10.3)	0.837
Spindle cells—Marked, *n* (%)	37 (37.8)	34 (91.9)	3 (8.1)	
Necrosis (+), *n* (%)	30 (30.6)	26 (86.7)	4 (13.3)	0.489
Cellular atypia—Mild, *n* (%)	27 (27.6)	25 (92.6)	2 (7.4)	
Cellular atypia—Moderate, *n* (%)	42 (42.9)	37 (88.1)	5 (11.9)	0.915
Cellular atypia—Severe, *n* (%)	29 (29.6)	26 (89.7)	3 (10.3)	
Erythrocyte extravasation (+), *n* (%)	83 (84.7)	76 (91.6)	7 (8.4)	0.179
Inflammation—Mild, *n* (%)	27 (27.6)	25 (92.6)	2 (7.4)	
Inflammation—Moderate, *n* (%)	44 (44.9)	38 (86.4)	6 (13.6)	1.00
Inflammation—Severe, *n* (%)	27 (27.6)	25 (92.6)	2 (7.4)	
Hyaline globules (+), *n* (%)	20 (20.4)	18 (90)	2 (10)	1.00

IQR, interquartile range; FFPE, formalin-fixed, paraffin-embedded.

**Table 4:** HHV-8 PCR results and internal control characteristics in KS and control groups.

Variable	PCR-Positive KS (*n* = 88)	PCR-Negative KS (*n* = 10)	Controls (*n* = 30)
HHV-8 Ct value, median (IQR)	20.92 (18.26–22.85)	Not detected	Not detected
Internal control Ct, median (IQR)	16.54 (14.69–17.91)	17.48 (13.62–20.82)	16.52 (15.12–18.57)
Adequate internal control amplification, *n* (%)	88 (100)	10 (100)	30 (100)
HHV-8 immunohistochemical expression, median % (IQR)	70 (60–80)	70 (60–80)	NA

All histopathologically confirmed KS cases showed LANA-1 positivity, whereas all control cases were LANA-1 negative. All PCR-negative KS samples showed adequate internal control amplification, suggesting that PCR negativity was unlikely to be caused by complete amplification failure.

**Table 5:** Diagnostics performance of HHV-8 PCR in FFPE Kaposi sarcoma biopsies.

Parameter	%	95% CI
Sensitivity	89.8	81.9–94.8
Specificity	100	88.6–100
Positive predictive value	100	95.8–100
Negative predictive value	75.0	59.7–86.8
Accuracy	92.2	86.2–96.0
False positive rate (1—specificity)	0	-
False negative rate (1—sensitivity)	10.2	-

False positive rate = 1—specificity; False negative rate = 1—sensitivity. These values are not independent criteria but are directly derived from sensitivity and specificity. CI: confidence interval.

**Table 6:** Agreement between HHV-8 PCR and histopathological diagnosis.

Histopathology	PCR Negative	PCR Positive	Total
Kaposi sarcoma (+)	10	88	98
Kaposi sarcoma (−)	30	0	30
Total	40	88	128

Data are presented as counts. κ = 0.34, *p* < 0.001.

## Data Availability

Data supporting the findings of this study are available from the corresponding author: Cemal Çiçek, upon reasonable request.

## Abbreviations

The following abbreviations are used in this manuscript:

CI	Confidence interval
Ct	Cycle threshold
DNA	Deoxyribonucleic acid
FFPE	Formalin fixed paraffin embedded
H&E	Hematoxylin and eosin
HHV-8	Human herpesvirus 8
HIV	Human immunodeficiency virus
IQR	Interquartile range
IL-6	Interleukin-6
KS	Kaposi sarcoma
LANA-1	Latency-associated nuclear antigen 1
NPV	Negative predictive value
PCR	Polymerase chain reaction
PPV	Positive predictive value
vGPCR	Viral G protein-coupled receptor
